# Metabolomic Characterization of Pediatric Acute-Onset Neuropsychiatric Syndrome (PANS)

**DOI:** 10.3389/fnins.2021.645267

**Published:** 2021-05-28

**Authors:** Federica Murgia, Antonella Gagliano, Marcello G. Tanca, Noga Or-Geva, Aran Hendren, Sara Carucci, Manuela Pintor, Francesca Cera, Fausto Cossu, Stefano Sotgiu, Luigi Atzori, Alessandro Zuddas

**Affiliations:** ^1^Clinical Metabolomics Unit, Department of Biomedical Sciences, University of Cagliari, Cagliari, Italy; ^2^Child and Adolescent Neuropsychiatry Unit, Department of Biomedical Sciences, University of Cagliari, Cagliari, Italy; ^3^Child and Adolescent Neuropsychiatry Unit, “A. Cao” Peditric Hosptal, “G. Brotzu” Hospital Trust, Cagliari, Italy; ^4^Interdepartmental Program in Immunology, Department of Neurology and Neurological Sciences, Stanford University School of Medicine, Stanford, CA, United States; ^5^Faculty of Health and Medical Sciences, University of Surrey, Guildford, United Kingdom; ^6^Paediatric Clinic, “A. Cao” Hospital, Cagliari, Italy; ^7^Child Neuropsychiatry Unit, Department of Medical, Surgical and Experimental Sciences, University of Sassari, Sassari, Italy

**Keywords:** metabolomics, pediatric acute-onset neuropsychiatric syndrome, neuroinflammation, oxidative stress, biomarkers

## Abstract

**Introduction:**

PANS is a controversial clinical entity, consisting of a complex constellation of psychiatric symptoms, adventitious changes, and expression of various serological alterations, likely sustained by an autoimmune/inflammatory disease. Detection of novel biomarkers of PANS is highly desirable for both diagnostic and therapeutic management of affected patients. Analysis of metabolites has proven useful in detecting biomarkers for other neuroimmune-psychiatric diseases. Here, we utilize the metabolomics approach to determine whether it is possible to define a specific metabolic pattern in patients affected by PANS compared to healthy subjects.

**Design:**

This observational case-control study tested consecutive patients referred for PANS between June 2019 to May 2020. A PANS diagnosis was confirmed according to the PANS working criteria (National Institute of Mental Health [NIMH], 2010). Healthy age and sex-matched subjects were recruited as controls.

**Methods:**

Thirty-four outpatients referred for PANS (mean age 9.5 years; SD 2.9, 71% male) and 25 neurotypical subjects matched for age and gender, were subjected to metabolite analysis. Serum samples were obtained from each participant and were analyzed using Nuclear Magnetic Resonance (NMR) spectroscopy. Subsequently, multivariate and univariate statistical analyses and Receiver Operator Curves (ROC) were performed.

**Results:**

Separation of the samples, in line with the presence of PANS diagnosis, was observed by applying a supervised model (R^2^X = 0.44, R^2^Y = 0.54, Q^2^ = 0.44, *p*-value < 0.0001). The significantly altered variables were 2-Hydroxybutyrate, glycine, glutamine, histidine, tryptophan. Pathway analysis indicated that phenylalanine, tyrosine, and tryptophan metabolism, as well as glutamine and glutamate metabolism, exhibited the largest deviations from neurotypical controls.

**Conclusion:**

We found a unique plasma metabolic profile in PANS patients, significantly differing from that of healthy children, that suggests the involvement of specific patterns of neurotransmission (tryptophan, glycine, histamine/histidine) as well as a more general state of neuroinflammation and oxidative stress (glutamine, 2-Hydroxybutyrate, and tryptophan-kynurenine pathway) in the disorder. This metabolomics study offers new insights into biological mechanisms underpinning the disorder and supports research of other potential biomarkers implicated in PANS.

## Introduction

Pediatric acute-onset neuropsychiatric syndrome (PANS) is a clinically heterogeneous disorder first described in 2012 after the modification of the Paediatric Autoimmune Neuropsychiatric Disorders Associated with Streptococcal infections (PANDAS) criteria (Swedo et al., 1998). The major clinical features of PANS consist of an acute-onset obsessive-compulsive disorder and/or severe eating restrictions, with at least two concomitant cognitive, behavioral, or affective symptoms such as anxiety, irritability, or depression (Swedo et al., 2012; Frankovich et al., 2015). These children experience neuropsychiatric symptoms in temporal association not only with GAS infection, but also with exposure to a wide variety of other infections and environmental or metabolic changes; thusly, the PANDAS diagnosis fell under the newly established umbrella category PANS (Swedo et al., 2012). The gender ratio is around 2:1, and symptoms usually commence in early childhood (7.3 ± 2.7 years). Clinical presentations were similar across sites, with all children presenting with acute-onset OCD symptoms and a constellation of other symptoms and disorders, including separation anxiety (86–92%), school issues (75–81%), sleep disruptions (71%), tics (60–65%), urinary symptoms (42–81%), and others (Swedo et al., 2015). As is the case with PANDAS, PANS is thought to be sustained by immune-mediated mechanisms (Swedo et al., 2015; Spinello et al., 2016), specifically those of so-called molecular mimicry occurring when pathogenic microorganisms express an antigenic structure indistinguishable (in terms of the amino acid sequence or three-dimensional structure) from self-antigens.

Under this rationale, the initiation of the symptomatic state results from an event (e.g., Group A Streptococcal infection) causing a localized immune response in the central nervous system (CNS), and the chronic relapsing course is due to the persistence of the immunological imbalance even after the resolution of the acute phase of the infection (Swedo et al., 2015). In PANS, various aetiological agents, including viruses (Hoekstra et al., 2005), Mycoplasma pneumonia (Müller et al., 2004), and Haemophilus influenza, supposedly act as triggers for activation of the immune response, together with the consequent release of chemical mediators of inflammation at the CNS level (Swedo et al., 2012; Frankovich et al., 2015). Structural and Functional abnormalities of the cortico-basal ganglia circuitry, similar to that seen in those with acute Sydenham’s Chorea, have been described in PANDAS. In particular, an enlarged striatal volume (Giedd et al., 2000; Elia et al., 2005) and an inflammatory state of the striatum, confirmed by positron emission tomography using a marker of microglial activation (Kumar et al., 2015), has been reported in PANDAS. A recent diffusion-weighted magnetic resonance imaging study identified cerebral microstructural differences in children with PANS in multiple brain structures (including deep gray matter structures such as the thalamus, basal ganglia, and amygdala) putatively related to a neuroinflammatory state (Zheng et al., 2020). In cohorts of rigorously selected subjects with PANS, evidence of post-infectious autoimmune processes and/or a condition of neuroinflammation was observed in over 80% of cases (Swedo et al., 2017).

A very recent paper showed that antibodies from children with PANDAS bind specifically to striatal cholinergic interneurons and alter their activity, sustaining the pathophysiology of rapid-onset obsessive-compulsive symptoms (Xu et al., 2021). Treatment of PANS involves different approaches: antibiotics to remove the potential source of neuroinflammation, anti-inflammatory and immunomodulatory treatments to regulate the immune system, and psychiatric medications to provide symptomatic relief (Swedo et al., 2015).

Despite this accumulating evidence, PANS is still regarded as a controversial clinical entity, consisting of a complex combination of psychiatric symptoms and adventitious changes and the expression of various serological variables of an autoimmune/inflammatory disease (Gagliano et al., 2020). In this scenario, research for new specific biomarkers is strongly desirable.

Metabolomics allows the simultaneous and relative quantification of various metabolites within a given biological sample (Mamas et al., 2011) through the application of two sensitive and specific methodologies, Nuclear Magnetic Resonance (NMR) (Smolinska et al., 2012) and Mass Spectrometry (MS) (Dettmer et al., 2007). By systematically identifying and quantifying the small molecule profile of a tissue or biofluid sample, known as “the metabolome,” metabolomics is thought to directly reflect the biochemical activity in a given organism or biological sample at a specific point in time. In the last decade, attempts have been made to use metabolomics to identify biomarkers of the various CNS disorders (Quinones and Kaddurah-Daouk, 2009; Hatano et al., 2016; Murgia et al., 2017; Pedrini et al., 2019); for example levels of taurine, succinate and other molecules like glycine and β-alanine were elevated in autistic subjects compared to controls (Glinton and Elsea, 2019). Currently, there are no systematic metabolomics datasets for PANS patients. A single case-study of a 10-year-old girl with PANS showed several metabolic pathways related to dysbiosis of microbial activity, protein biosynthesis, and amino acid metabolism (Piras et al., 2020).

The present study aims to identify specific serum metabolomic profiles in a sample of children clinically diagnosed with PANS, measured through ^1^H-NMR spectroscopy.

## Materials and Methods

### Study Population and Inclusion Criteria

PANS patients referred to the outpatient clinics of Child and Adolescent Neuropsychiatric Unit “G. Brotz” Hospital Trust, Cagliari (June 2019 to May 2020), were enrolled in this observational study.

An extensive physical, neurological, and psychiatric examination was performed. Moreover, a clinical laboratory test, consisting of a complete blood count, renal and liver function tests, mineral panel, thyroid indices, and inflammation blood markers, was undertaken to exclude metabolic concomitants or systemic diseases. All participants were assessed by a panel of standardized scales and questionnaires encompassing the Pediatric Acute Neuropsychiatric Symptom Scale (PANSS) (Pandas Network, 2018) to screen the symptoms and their severity. Details of the PANSS scale are reported in Supplementary Materials.

Diagnosis of PANS was confirmed by two child psychiatrists (AG and FC), according to the PANS working criteria defined by experts convened at the National Institute of Mental Health (NIH) in July 2010 (Swedo et al., 2012):

(I) Abrupt dramatic onset of OCD or severely restricted food intake;
(II) Concurrent presence of additional neuropsychiatric symptoms (with similarly severe and acute onset), from at least two of the following seven categories:

(1) Anxiety;
(2) Emotional lability and/or depression;
(3) Irritability, aggression, and/or severely oppositional behaviors;
(4) Behavioral (developmental) regression;
(5) Deterioration in school performance (related to attention-deficit/hyperactivity disorder [ADHD]-like symptoms, memory deficits, and cognitive changes);
(6) Sensory or motor abnormalities;
(7) Somatic signs and symptoms, including sleep disturbances, enuresis, or urinary frequency;

(III) Symptoms not better explained by a known neurological or medical disorder, such as Sydenham’s Chorea.

Exclusion criteria were the following: (I) occurrence of immunological diseases or cancer; (II) presence of other medical or neurological/psychiatric diseases; (III) active treatment with psychoactive substances, non-steroidal anti-inflammatory drugs or corticosteroid agents; (IV) patients’ unwillingness to participate in the study.

Among a total of 52 consecutive outpatients referred for PANS, 34 met the inclusion criteria of this study. The mean age at recruitment was 9.5 years. (SD 2.9); the gender ratio was 10/24 (71% male). The serum samples of the affected patients were compared to the serum of 25 neurotypical subjects matched for age and gender (mean age 12.12 years.; SD 2.1, 64% male), and living in the same geographic area as the clinical group. The control group encompassed children with no autoimmune pathologies, neurodevelopmental and psychiatric disorders and with adequate academic achievement and functional performance. The aim of the study was to compare the metabolic profile of the two classes of patients, considering both a model including all of the samples together and models considering males and females separately, to underline possible differences based on gender.

The study was conducted with the approval of the independent Ethical Committee of Cagliari University Hospital (Prot. PG/2019/7413 on 29/05/2019). All the parents and all children older than 12 were given a full explanation of the study’s method and purpose. The parents signed the consent form, agreeing to participate, and to the data being published. Furthermore, the research was conducted in accordance with the Declaration of Helsinki, V edition (2000).

### Sample Preparation for ^1^H-NMR

Ten milliliter of blood were collected from each subject and were centrifuged at 2,500 *g* for 10 min at 4°C. The obtained serum samples were stored at −80°C until analysis. Subsequently, samples were thawed and treated with a modified Folch method (Bligh and Dyer, 1959; Lorefice et al., 2019) to extract and separate hydrophilic and lipophilic metabolites. 400 μL of each serum sample were mixed with 600 μL of methanol, 600 μL of chloroform, and 175 μL of Milli-Q water. The samples were vortexed for 1 min and centrifuged for 30 min at 1700 *g* at room temperature. Aliquots (10 μL) from each sample were used to create a pool for quality control (QC) samples. The QC samples were analyzed at the beginning and the end of the analysis. The hydrophilic and hydrophobic phases were obtained. The water-phase was divided into two aliquots and concentrated overnight using a speed vacuum centrifuge.

### ^1^H-NMR Analysis

For the ^1^H-NMR analysis, 700 μL of the water-phase containing low-weight molecules (amino acids, sugars, etc.) for each sample were concentrated overnight in a speed-vacuum. The concentrated water-phase was resuspended in 690 μL of D_2_O phosphate buffer (pH 7.4) and 10 μL trimethylsilyl propanoic acid (TSP) 5.07 mM. TSP was added to provide an internal reference for the chemical shifts (0 ppm). A total of 650 μL of the solution was transferred to a 5 mm NMR tube.

The samples were analyzed with a Varian UNITY INOVA 500 spectrometer (Agilent Technologies, Inc., Santa Clara, CA, United States), which was operated at 499 MHz and equipped with a 5 mm triple resonance probe with z-axis pulsed field gradients and an auto-sampler with 50 locations. One-dimensional ^1^H-NMR spectra were collected at 300 K with a pre-sat pulse sequence to suppress the residual water’s signal. The spectra were recorded with a spectral width of 6,000 Hz; a frequency of 2 Hz; an acquisition time of 1.5 s; a relaxation delay of 2 ms; and a 90° pulse of 9.5 μs. The number of scans was 256. Each Free Induction Decay (FID) was zero-filled to 64 k points and multiplied by a 0.5 Hz exponential line broadening function. The spectra were manually phased and baseline corrected. Using MestReNova software (version 8.1, Mestrelab Research SL), each ^1^H-NMR spectrum was divided into consecutive “bins” of 0.04 ppm. The spectral area investigated was the region between 0.6 and 8.6 ppm. The regions between 4.60 and 5.2 ppm and between 5.24 and 6.6 ppm were excluded to remove variations in the pre-saturation of the residual water resonance and spectral regions of noise. To minimize adverse effects resulting from the serum samples’ differing concentrations, the integrated area within each bin was normalized to a constant sum of 100. The final data set consisted of a 150 × 59 matrix. The columns represent the normalized area of each bin (variables), and the rows represent the samples (subjects).

### Multivariate Statistical Analysis

Multivariate statistical analysis was performed on ^1^H-NMR data with SIMCA-P software (ver.15.0, Sartorius Stedim Biotech, Umea, Sweden; (Eriksson et al., 2013). The variables were Pareto scaled to emphasize all metabolite signals and reduce the spectral noise for the ^1^H-NMR analysis.

The initial data analyses were conducted using Principal Component Analysis (PCA), which is used to explore the sample distributions without classification. In particular, PCA analysis was performed to observe intrinsic clusters and find outliers. For this aim, the DmodX and Hotelling’s T2 tests were applied. The PCA model was performed including the QC samples to corroborate the quality of the analysis.

Orthogonal Partial Least Square Discriminant Analysis (OPLS-DA) was subsequently applied. OPLS-DA maximizes the discrimination between samples assigned to different classes, in this case discriminating between patients with PANS and healthy subjects. OPLS-DA was also used to perform a gender analysis. In particular, pathological male and female subjects were separately compared to the matched controls. The aim of this analysis was to find specific metabolic features to define gender-related differences in the PANS profile which could have influenced the final result. The OPLS-DA model removes variability not relevant to class separation (Rousseau et al., 2008). The variance and the predictive ability (R^2^X, R^2^Y, Q^2^) were established to evaluate the suitability of the models. An additional permutation test (*n* = 400) was performed to validate the model. This rigorous test compares the fit of the original model with that of randomly permuted models (Lindgren et al., 1996). In particular, the permutation test evaluates model validity in terms of the explained variance parameter (R^2^) and the cross-validation parameter (Q^2^), that indicates fit and accuracy of the prediction, respectively. A CV-ANOVA (analysis of variance testing of cross-validated predictive residuals) test was performed simultaneously to establish the significance of the OPLS-DA model (*p* < 0.05). Through the multivariate analysis it was also possible to investigate a potential linear relationship between the metabolic profile (predictor variables, e.g., metabolites) and the clinical parameters such as dependent variables like age and scale of severity for PANS. For this aim, PLS projection to latent structure regression models were performed.

Variables corresponding to a VIP (Variables Important in the Projection) value of > 1 (a measure of their relative influence on the model) from the OPLS-DA model, together with the relative S-plot, were selected as the most important. Indeed, VIPs of > 1 are the most relevant for explaining Y (assignment of two classes). The selected variables were identified using the Chenomx NMR Suite 7.1 (Chenomx Inc., Edmonton, Alberta, Canada; Weljie et al., 2006). GraphPad Prism software (version 7.0, GraphPad Software, Inc., San Diego, CA, United States) was used to perform the univariate statistical analysis of the data. To verify the significance of the metabolites resulting from multivariate statistical analysis, the U-Mann Whitney test was performed, followed by ROC curves to test the sensitivity and specificity of the metabolites with *p*-values < 0.05. ROC curves are conventionally used to evaluate diagnostic performance in clinical research. Moreover, Pearson correlation between the clinical parameters (age and PANSS scale) was performed for each significant metabolite.

### Pathway Analysis

To help researchers identify the most relevant pathways involved in the conditions under study, metabolic pathways were generated using MetaboAnalyst 5.0 (Chong et al., 2019), a web server designed to obtain metabolomic data analysis, visualization, and biological interpretation^1^. In particular, the pathway analysis module of MetaboAnalyst 5.0 helps researchers identify the most relevant pathways involved in the conditions under study using the high-quality Kyoto Encyclopedia of Genes and Genomes (KEGG) metabolic pathways as the backend knowledgebase. The Pathway Analysis module combines results from powerful pathway enrichment analysis with pathway topology analysis.

## Results

### The Metabolic Signature of PANS Patients Is Distinct From Healthy Controls

**FIGURE 1:**
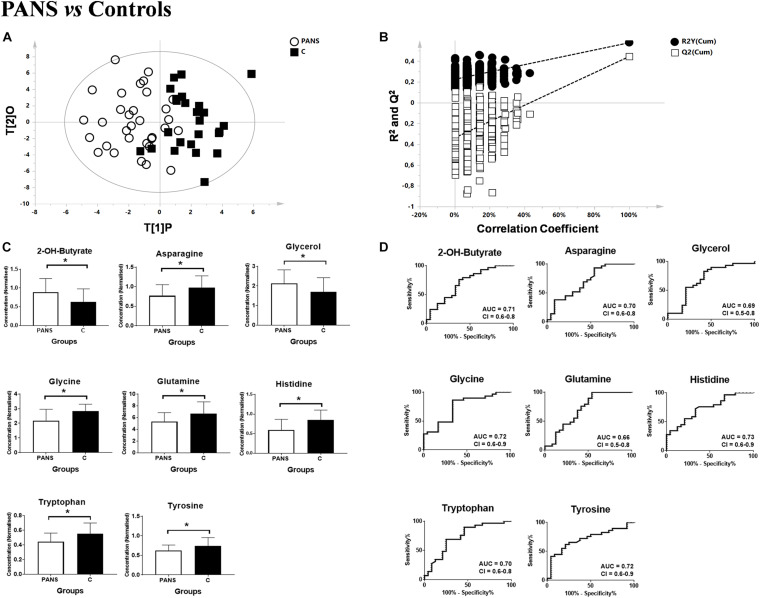
Comparison between PANS and Controls patients. **(A,B)** OPLS-DA models of the analyzed classes. PANS (white circles) vs. Controls subjects (black boxes) with the respective permutation test. **(C)** Bar graphs and **(D)** ROC curves of the metabolites exhibiting a *p*-value of < 0.05. U-Mann Whitney analysis was used, and subsequently Holm-Bonferroni correction was applied. White bars represent the PANS class while black bars represent the control patients. *p < 0.05.

**FIGURE 2:**
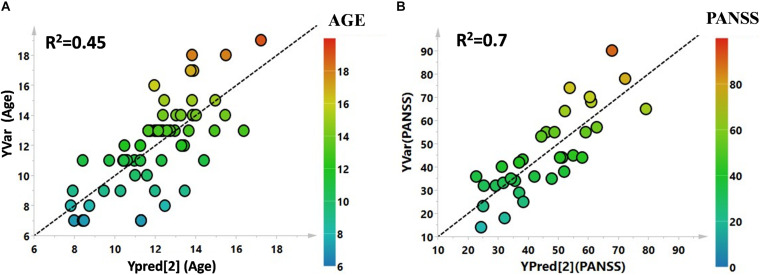
**(A)** PLS correlation analysis between the metabolic profile of the enrolled subjects and the age. **(B)** PLS correlation analysis between the metabolic profile of the affected patients and the PANSS severity score.

Utilizing ^1^H-NMR analysis of serum samples, we identified a total of 44 hydrophilic metabolites (Supplementary Figure 1). Each of these metabolites, which were quantified for each patient, were organized in a matrix to undergo multivariate analysis. Firstly, non-supervised multivariate Principal Component Analysis (PCA) was performed using the bins dataset. The aim of this approach was to identify any outlying samples or any possible sample cluster without previous classification. The obtained score plot and the result of the Hotelling’s T^2^ test did not identify any outliers (data not shown). A separation of the samples, in line with the presence of PANS diagnosis, was subsequently observed after applying the supervised OPLS-DA model (Figure 1A). These results were then validated using the respective permutation test (Figure 1B). The statistical parameters of the model were: R^2^X = 0.44, R^2^Y = 0.57, Q^2^ = 0.44, *p* < 0.0001. The cumulative values of total Y-explained variance (R^2^) and the Y-predictable variation (Q^2^) values of the permutation test indicated proper modeling (Intercept R^2^\Q^2^ = 0.17/−0.28). The same patient classification was used to construct the supervised model considering males and females separately (Supplementary Figure 2). The aim of this analysis was to define possible gender differentials in metabolite concentrations which could have influenced the result. A good separation was also observed in these models, and we further observed that the metabolites responsible for the separation were the same as those causing separation in the complete model. Moreover, demographic data such as age, and the phenotypic severity were correlated with the total metabolic profile. A weak correlation was found between age and the metabolic profile (R^2^ was equal to 0.52) while a strong correlation was found between the phenotypic severity parameters express as PANSS scale evaluation and the metabolic profile (R^2^ was equal to 0.7). The results are shown in Figures 2A,B.

### Metabolite Analysis Highlights Energy and Neurotransmission Pathways, Corresponding to the Clinical Symptomology of PANS

Subsequently, the most significant variables were identified through analysis of the S-plot and through the corresponding VIP-value of the complete supervised model. Variables with a VIP-value of > 1 were identified with Chenomx and underwent univariate statistical analysis with the U-Mann Whitney test. A subsequent Holm-Bonferroni correction for multiple comparisons was applied.

**FIGURE 3:**
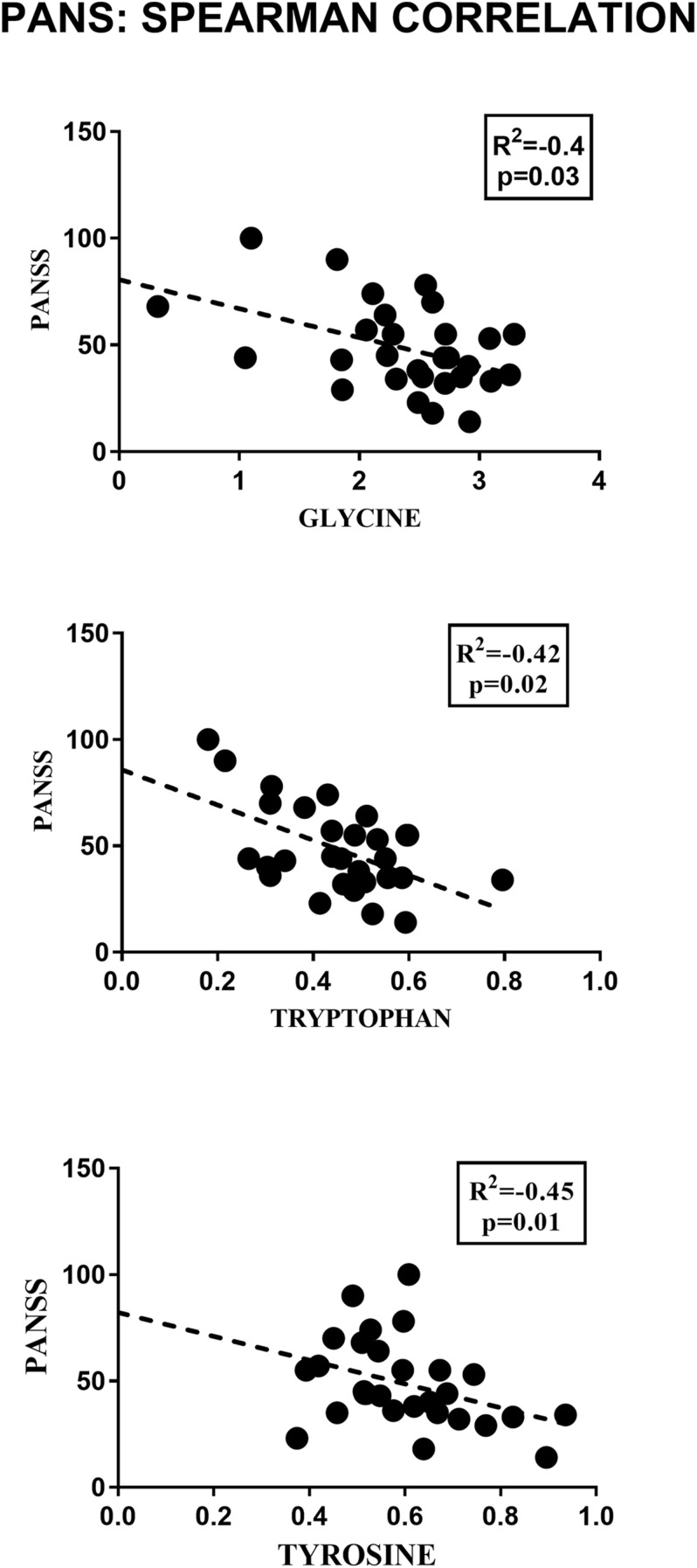
Pearson correlation analysis between the concentrations of the significant metabolites and the PANSS severity score. Only glycine, tryptophan and tyrosine showed a significant correlation with the clinical parameter considered.

2-Hydroxybutyrate and glycerol were found to be increased in PANS patients, while asparagine, glycine, glutamine, histidine, tryptophan, tyrosine were found to be decreased. These metabolites exhibited the most significant differences between PANS and Controls, denoted by a *p*-value of < 0.05 corrected for multiple comparisons (Holm-Bonferroni test, Figure 1C), and were selected to create ROC curves (Figure 1D); the corresponding statistical parameters are reported in Table 1. Considering the result of the PLS correlation analysis, we decided to test the correlation between the PANSS scale and each significantly altered metabolite in the PANS class compared to the control subjects by conducting Pearson correlation analysis. Only glycine (*p* = 0.03), tryptophan (*p* = 0.02) and tyrosine (*p* = 0.01) were found to be significantly correlated with the severity of the syndrome (Figure 3).

**TABLE 1:** Statistical parameters of the univariate analysis from the comparisons between PANS and Controls.

**Metabolites**	**PANS**	***p*-value**	***p*-value corrected**	**ROC-CURVE**			
				**AUC**	**Std. Er**	**CI**	***p*-value**
**Serum PANS vs. Control**							
2-OH-Butyrate	**+**	0.01	0.05	0.71	0.07	0.6–0.8	0.01
Acetone	**+**	0.04	0.09	0.66	0.07	0.5–0.8	0.04
Alanine	**−**	0.04	0.09	0.66	0.07	0.5–08	0.04
Asparagine	**−**	0.01	0.05	0.70	0.07	0.6–0.8	0.01
Dimethylamine	**−**	0.04	0.09	0.66	0.07	0.5–0.8	0.04
Glycerol	**+**	0.01	0.05	0.69	0.07	0.5–0.8	0.01
Glycine	**−**	0.001	0.01	0.72	0.06	0.6–0.9	0.002
Glutamine	**−**	0.008	0.05	0.71	0.07	0.5–0.8	0.01
Histidine	**−**	0.003	0.03	0.73	0.06	0.6–0.9	0.003
Isoleucine	**−**	0.03	0.09	0.67	0.07	0.5–0.8	0.03
Tryptophan	**−**	0.003	0.03	0.73	0.07	0.6–0.8	0.004
Tyrosine	**−**	0.006	0.04	0.72	0.07	0.6–0.8	0.007

*U-Mann Whitney test, Holm-Bonferroni correction for multiple comparisons and ROC curves were performed.*

### Pathway Enrichment Indicates the Involvement of Inflammatory and Neurotransmitter Pathways in PANS

**FIGURE 4:**
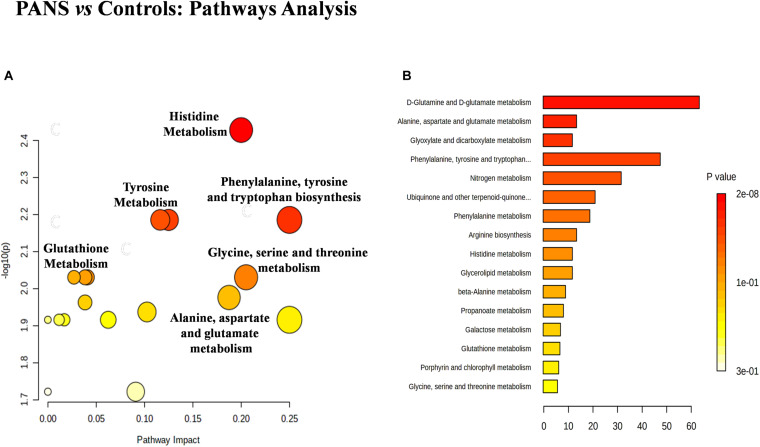
The metabolic pathways most altered in patients with PANS diagnosis were histidine metabolism, phenylalanine, tyrosine and tryptophan metabolism, tyrosine metabolism, glutathione metabolism, glycine, serine and threonine metabolism, alanine, aspartate and glutamate metabolism, glutamine and glutamate metabolism. **(A)** The size of the circles represent the pathway impact while the colors (varying from yellow to red) reflect the different levels of significance. **(B)** Also the enrichment analysis performed with the same software, confirm the same altered metabolic nets.

The discriminant metabolites of the comparison between PANS and controls which passed the Holm-Bonferroni correction (2-hydroxybutyrate, asparagine, glutamine, glycerol, glycine, histidine, tryptophan, and tyrosine) were used to perform the pathways analysis and the enrichment analysis. MetaboAnalyst was used to characterize the altered pathways in the PANS group. Compared to Controls, the most altered pathways that we considered for the interpretation of the data were histidine metabolism, phenylalanine, tyrosine and tryptophan metabolism, glutathione metabolism, glycine, serine and threonine metabolism, alanine, aspartate and glutamate metabolism, glutamine, and glutamate metabolism, according to the parameters suggested by the software including pathway impact and *p*-value (Figure 4).

## Discussion

The metabolomics approach applied in this study allowed the identification of a set of hydrophilic metabolites that appears to represent a specific pattern characterizing the PANS condition. This pattern was independent of the gender of the patients, as demonstrated by our results. Moreover, through the statistical tools employed by this study we can hypothesize that the age of the enrolled subjects weakly correlate with changes in metabolic profile, while a significant correlation was found between the severity scale of PANS and the metabolic profile of the affected patients. The current lack of reliable biomarkers for PANS leads us to consider our data as an important starting point in understanding the pathological features of this complex syndrome. Metabolomics profiles are considered the omics layer most proximal to phenotype (Patti et al., 2012; Haas et al., 2017): In contrast to genetic variants, metabolomics profile can be either a cause or a consequence of the phenotype of interest. Following this reasoning, analysis of the putative role of specific metabolites and altered pathways may help to define a comprehensive metabolic pattern of the disorder, which, in turn, may be of help in determining specific biomarkers.

The amino acid tryptophan (Trp) was found at significantly lower serum concentrations in patients with PANS than in controls. More interestingly, though, this metabolite also negatively correlated with the severity of the syndrome as demonstrated by the result of the Person correlation. The pathways analysis confirmed an alteration in Trp metabolism. In recent years, Trp metabolism has been increasingly recognized as central to the pathogenesis of many neuropsychiatric disorders. In particular, a decrease in Trp level was found in major depressive disorder (Moreno et al., 2010; Liu et al., 2015), schizophrenia (Yao et al., 2010; Xuan et al., 2011) and exhaustion disorder (Hadrévi et al., 2019). Our findings are in line with these reports, suggesting that Trp metabolism may be common to several neuropsychiatric conditions that share several clinical features with PANS, such as irritability, anxiety, emotional lability and thought disturbances.

The predominant pathway of Trp metabolism in humans is the Kynurenine Pathway (KP) (Dantzer, 2017): several tryptophan-kynurenine pathway metabolites have been increasingly recognized as crucial facets immuneregulatory mechanisms, neuroinflammation (Mándi and Vécsei, 2012) and microglial activation, potentially related to severe neuropsychiatric disorders (Horikawa et al., 2010) such as depression (Dantzer, 2017), psychosis and Autism Spectrum Disorder (ASD) (Olloquequi et al., 2018).

This putative mechanism is supported by studies on animal models; symptoms such as severe involuntary movement, increased locomotor activity and persistently impaired active avoidance learning, suggestive of a PANS-like phenotype, can be induced by administering quinolinic acid (QA), a potently neurotoxic metabolite of the KP, into the striatum (Vécsei and Flint Beal, 1991).

As well as products of the KP pathway, tryptophan is also the precursor to 5-hydroxytryptamine (5-HT) (Wichers and Maes, 2004). Given this, it is reasonable to assume a direct link between the two biomolecules. 5-HT has diverse roles in memory, mood, anxiety, aggression, pain, sleep, and eating behavior (Sandyk, 1992). Accordingly, 5-HT deficit has been implicated in several neuropsychiatric illnesses (Bell et al., 2001), including major depressive disorder, obsessive-compulsive disorder, and anxiety (Baumgarten and Grozdanovic, 1998; Colle et al., 2020). Interestingly, several conditions involving Trp malabsorption (e.g., Hartnups disease) are often characterized by symptoms such as psychosis or depression ^(^Oyanagi et al., 1967), as well as dietary Trp deprivation having been shown to exacerbate ASD symptoms (McDougle, 1996). Moreover, serotonin is the major substrate required for melatonin synthesis, and consequently, serotonin deficiency reduces the synthesis of melatonin (Zimmermann et al., 1993). This suggests a possible explanation for the sleep disruption frequently occurring in PANS, described as parasomnias (nightmares, nocturnal pavor, sleepwalking or somnambulism), as well as difficulties in initiating or maintaining sleep (early or intermediate insomnia), early awakenings (terminal insomnia), REM sleep disorders such as REM Sleep Without Atonia (RSWA), and sleep movement disorders such as Periodic Limb Movement Disorder (PLMD) (Santoro et al., 2018). Combined, these data strongly suggest that abnormal tryptophan metabolism may play a role in the pathophysiology of PANS since the presentation of PANS symptoms encompasses tics and anxiety, as well as obsessive-compulsive and psychotic symptoms.

Glycine is another metabolite significantly decreased in concentration (*p* = 0.0048) in the serum of patients with PANS compared to healthy controls. The potential pivotal role of this metabolite in PANS was also confirmed by its direct correlation with the severity scale of this pathological condition. Glycine’s biomolecular role is notable in several psychiatric disorders (Rujescu and Giegling, 2016; Erjavec et al., 2018; Humer et al., 2020). Glycine is widely distributed in the mammalian CNS, functioning as an inhibitory or excitatory neurotransmitter, depending on its localization. Glycine is the main neurotransmitter in inhibitory interneurons of the spinal cord, brainstem, and in other brain regions involved in the processing of sensorimotor information and locomotor behavior ^(^Chatterton et al., 2002). Glycine can activate two classes of distinct ligand-gated ion channels: chloride-permeable inhibitory GlyRs (Gielen et al., 2015) and cation-selective excitatory NMDARs. Electrophysiological, immunocytochemical, and *in-situ* hybridization studies have shown that GlyRs are prominent in the brainstem and spinal cord (Altschuler et al., 1986; Alvarez et al., 1997) and detectable also in the following brain regions: the prefrontal cortex, hippocampus, amygdala, hypothalamus, cerebellum, nucleus accumbens, ventral tegmental area, and substantia nigra (Ye et al., 1998; McCool and Botting, 2000; Chattipakorn and McMahon, 2002). As an excitatory neurotransmitter, glycine acts as a co-agonist of ionotropic NMDARs, allowing for depolarization, removal of the magnesium blockade, and Na^+^/Ca^2+^ passage through the ion channel, which ultimately enhances the glutamatergic excitatory tone that is critical for learning and neuronal plasticity (Nakazawa et al., 2004; Collingridge et al., 2013). Notably, the affinity of glycine for NMDARs is significantly higher than that of GlyRs (EC50 = 134 nM vs. EC50 = 270 mM) (Chattipakorn and McMahon, 2002; Cubelos et al., 2014). Thusly, under physiological conditions, endogenous glycine may exert a mainly excitatory effect in the hippocampus, where both GlyRs and NMDARs are expressed. Interestingly, an increase in serum glycine levels measured by ^1^H-NMR spectra has been shown to differentiate schizophrenia (with higher glycine levels) from bipolar patients (with glycine plasma levels similar to controls), suggesting an important role for glycine in regulating cognition (Tasic et al., 2019) and affective regulation, an observation parallelled in animal models of neuropsychiatric disorders (Humer et al., 2020). Irritability, emotional lability, behavioral regression has been shown to be a crucial component of the PANS clinical presentation (Gagliano et al., 2020), rendering the involvement of glycine metabolism in PANS biologically plausible. Remarkably, antibodies directed against extracellular receptors on the synaptic surface, such as the NMDA receptor, are known to lead to NMDA receptor hypofunction in patients, resulting in behavioral and psychotic manifestations in specific forms of autoimmune encephalitis (Endres et al., 2019).

Glutamine levels, and the activity of associated pathways (glutamine and glutamate metabolism and aspartate and glutamate metabolism), were also found to be lower in PANS patients compared to the neurotypical control group. Glutamine in the CNS plays an essential role in the glutamate/GABA-glutamine cycle; it is transferred from astrocytes to neurons, where it replenishes the inhibitory and excitatory neurotransmitter pools (Masdeu et al., 2016). Decreased glutamine plasma concentrations may be caused by several factors, including acute inflammatory activity and consecutive distribution abnormalities, and therefore is not, *per se*, an indicator of actual shortage (Leke and Schousboe, 2016). Glutamine is an essential nutrient in all rapidly proliferating cells, including immune cells; it provides many different building blocks for these cells and simultaneously maintains redox balance by providing reducing equivalents, which are also necessary to allow the appropriate functioning of the immune system. Glutamine supplementation may be beneficial in patients with a long-standing inflammatory activity that are not producing sufficient quantities of glutamine either due to malnutrition or because they cannot meet the demands of extremely severe inflammatory illnesses (e.g., in ICU patients). More precise information on glutamine’s role in PANS could reveal innovative therapeutic approaches.

Histidine, which was also found to be at a significant deficit in PANS patients, is a precursor of the ubiquitously distributed neurohormone neurotransmitter histamine. In the CNS, histamine is known to regulate sleep and wakefulness, learning and memory, feeding, and energy (He et al., 2012; Soeters and Grecu, 2012). A decrease in plasma histidine level has been considered a metabolomic signature of schizophrenia, although contrasting results have been reported on plasma and brain histamine levels (Hu and Chen, 2017). Taken together, the significant decrease of histidine observed in the present study indicates the need for further investigation on a putative role of histamine in the pathophysiology of PANS; histamine levels were not measured in the present study, but the increasing availability of histaminergic receptor modulators supports interest in this neurotransmitter.

Finally, 2-Hydroxybutyrate was found to have significantly increased concentration in the serum of PANS patients. This metabolite is typically produced as a result of excessive glutathione anabolism (Takahashi et al., 2006). Glutathione is an antioxidant whose synthesis has been demonstrated to undergo compensatory upregulation in the blood of individuals experiencing increases in oxidative stress, such as smokers (Gall et al., 2010), as well as in the aging brain (Agarwal et al., 2019). The 2-Hydroxybutyrate increase observed in this sample may indicate that oxidative stress may be considered a crucial feature of PANS.

The specific plasma metabolites observed in the present study might reflect particular changes in metabolic pathways induced by inflammation, blood-brain barrier breakdown, and dysregulation of energy metabolism. These processes may activate a common final pathway that can be triggered by different agents.

The results of the present study suggest unique plasma metabolite profiles in PANS patients, significantly differing from healthy children, showing abnormal levels of metabolites associated with neurotransmission (tryptophan, glycine, histamine/histidine) and generalized energy deficiency, oxidative stress, neuroinflammation (glutamine, 2-Hydroxybutyrate and, potentially, the tryptophan-kynurenine pathway). Overall, the evidence provided by the present study is consistent with the accumulating data supporting the presence of a neuroinflammatory component in several psychiatric illnesses (Tong et al., 2016). Inflammatory biomarkers have been found in common neuropsychiatric outcomes (Najjar et al., 2013), such as ADHD (Yuan et al., 2019), ASD (Dunn et al., 2019), bipolar disorder (Xu et al., 2015), depression (Muneer, 2016), and schizophrenia (Dowlati et al., 2010; Na et al., 2014).

However, the present work is not without limitations. We focused on a homogeneous group of children and adolescents diagnosed with PANS; a comparison with patients presenting a limited association of two or more PANS criteria, but not the complete disorder, can strengthen our study.

Further analysis of patients with PANS, using additional methods, such as Mass Spectrometry, may be able to identify additional metabolites that cannot be detected by our NMR approaches, such as lipophilic compounds.

More in-depth investigation on a larger cohort with additional tools is needed to confirm these findings and support their interpretation. Nevertheless, our results strongly suggest the utility of metabolomics as a means to enhance PANS biomarker research. We argue that serum metabolites can ultimately provide a map of the regulation of the metabolic pathways in the central nervous system of individuals with PANS. This is due to the syndrome exhibiting altered mechanistic biochemical patterns, linked to exposure to an environmental stimulus (e.g., infectious agents or stressors) with the capability of disrupting metabolism. This, in turn, results in changes that can be captured as metabolic signatures potentially providing information with regard to PANS pathophysiology.

## Conclusion

PANS is currently conceptualized as a complex syndrome with several etiologies and disease mechanisms, encompassing psychiatric symptoms and arising from inflammatory/immune abnormalities triggered by a variety of agents (Swedo et al., 2012; Frankovich et al., 2015). The specific plasma metabolites observed in the present study might reflect specific changes in metabolic pathways induced by inflammation, blood-brain barrier breakdown, and dysregulation of energy metabolism. These processes may activate a common final pathway that can be triggered by different agents. In conclusion, the results of the present study suggest unique plasma metabolite profiles in PANS patients, significantly differing from healthy children, showing abnormal levels of metabolites associated with neurotransmission (tryptophan, glycine, histamine/histidine) and generalized energy deficiency, oxidative stress neuroinflammation (glutamine, 2-Hydroxybutyrate and, potentially, the tryptophan-kynurenine pathway). Taken together, our results strongly suggest the utility of metabolomics as a means to enhance PANS biomarker research.

## Data Availability Statement

The raw data supporting the conclusions of this article will be made available by the authors, without undue reservation.

## Ethics Statement

The studies involving human participants were reviewed and approved by COMITATO ETICO INDIPENDENTE Azienda Ospedaliero Universitaria di Cagliari P.O. San Giovanni di Dio: via Ospedale 54 – 09124 Cagliari Segreteria Tecnico Scientifica tel. 0706092547–0706092262—fax 0706092262 Web: www.aoucagliari.it/home/it/comitato_etico_.page. Written informed consent to participate in this study was provided by the participants’ legal guardian/next of kin.

## Author Contributions

FM and AG provided substantial contributions to the conception and design of the work, the analysis and interpretation of data for the work, and drafting of the work. MT and AH provided substantial contributions to patient recruiting, database collection, and acquisition and analysis of data. FCe, MP, and FCo managed the patient recruiting and the database collection. SC and SS revised the work critically for important intellectual content. NO-G, LA, and AZ revised the work critically for important intellectual content and gave their final approval of the version to be published. All authors contributed to the article and approved the submitted version.

## Conflict of Interest

AG was on the advisory boards for Eli Lilly and Shire. She is/has been involved in clinical trials conducted by Eli Lilly, Shire, Lundbeck, Janssen, and Otsuka. She has been a speaker for Novartis, Eli Lilly, and Shire. SC was collaborating on projects from the European Union (7th Framework Program) and as a sub-investigator in sponsored clinical trials by Lundbeck Otsuka and Janssen Cilag. Travel support from Fidia Farmaceutici. AZ served in an advisory or consultancy role for Angelini, Lundbeck, Otsuka, and Edu-Pharma. He received conference support or speaker’s fee from Angelini, Otzuka, and Takeda. He is/has been involved in clinical trials conducted by Angelini, Roche, Lundbeck, Janssen, Servier, and Otsuka. He received royalties from Oxford University Press, and Giunti OS. SS received conference support or speaker’s fee from Bayer Pharma, Biogen Idec, Merck-Serono, Novartis, and Teva. He has been involved in clinical trials conducted by Bayer Pharma, Biogen and Teva. He received royalties from Documenta (Ed). The present work is unrelated to the above grants and relationships. The remaining authors declare that the research was conducted in the absence of any commercial or financial relationships that could be construed as a potential conflict of interest.

## Footnotes

1 www.metaboanalyst.ca
