# Supplementary material for: Metabolomic Characterization of Pediatric Acute-Onset Neuropsychiatric Syndrome (PANS)
Source: Front Neurosci. 2021 May 28;15:645267. doi: 10.3389/fnins.2021.645267 (PMC8194687; doi:10.3389/fnins.2021.645267)
Supplement: Supplementary file 1 [file Data_Sheet_1.pdf]

Supplementary materials

PANSS

The Pediatric Acute Neuropsychiatric Symptom Scale (PANSS) was developed, based on the clinical experience, by Swedo S, Kovacevic M, Latimer B and Leckman J, with the help of Pohlman D, Moore K and many other parents.

In our study, PANSS was used by investigators with parents of patients, rating the severity of symptoms and impairment, on the same day as the blood draw.

The structure of the scale provides for each symptom, included in the PANS criteria, a score from 0 to 5 points (none to very severe), with a total score range of 0–50 points.

The impairment in self-esteem, family life, social acceptance, and school or job functioning, is estimated with a total score range of 0–50 points (no impairment to extreme difficulties).

For the PANS scale, see:
https://pandasnetwork.org/wpcontent/uploads/2018/11/pandas_pans_scale.pdf

Acknowledgments

We thank Susan Swedo and James Leckman for their valuable advice and permission to use the PANS scale and all participating families for their contributions.

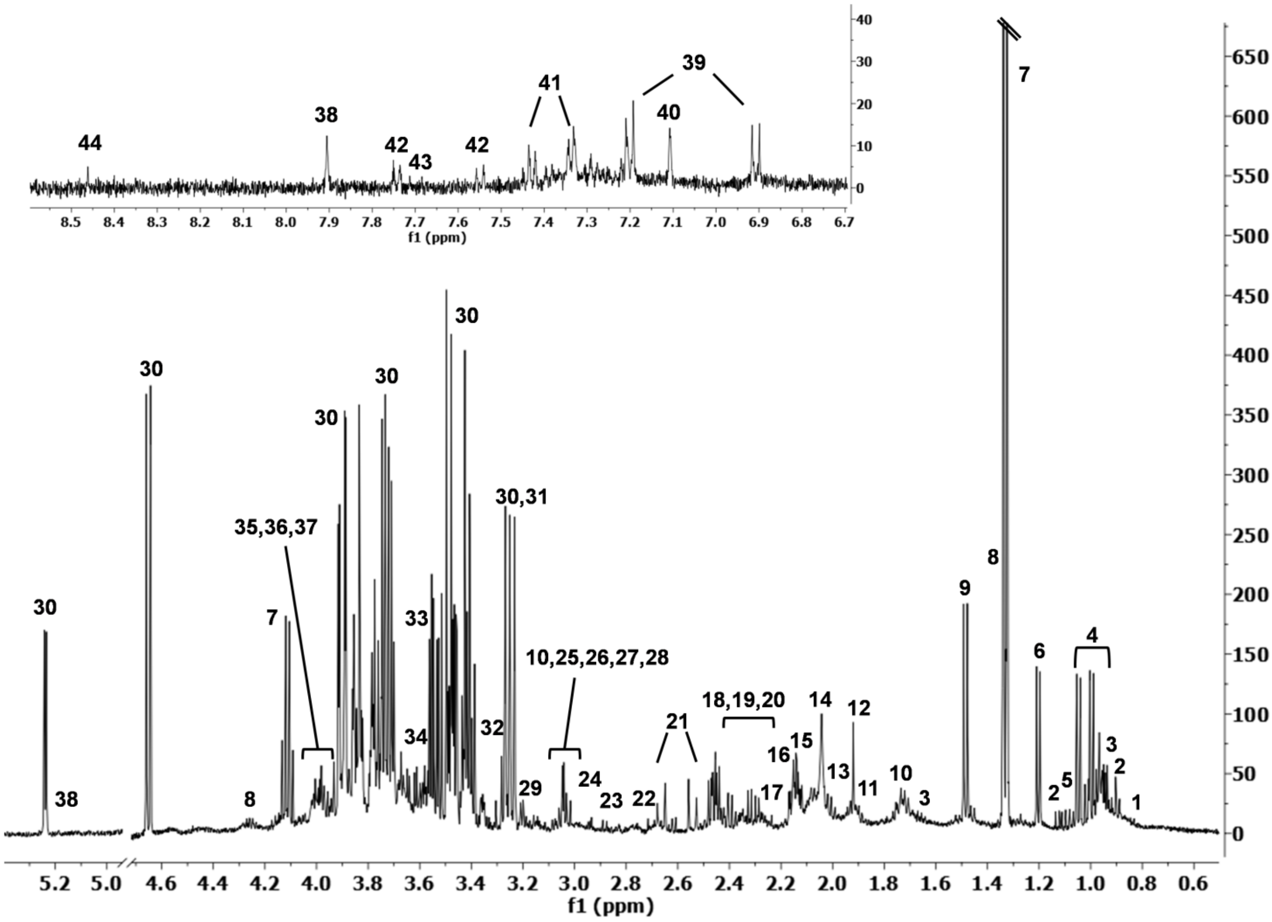

Figure S1 Signal's assignments of the serum metabolites in an ^{1}H-NMR spectrum: 1. 2-Hydroxyisovalerate; 2. 3-methyl-2-Oxoglutarate; 3. 2-Hydroxybutirate; 4.Branched Aminoacids: Valina, Leucine, Isoleucine, 5. 2-Methylglutarate; 6. 3-Hydroxybutirate; 7. Lactate; 8.Threonine; 9. Alanine; 10. Lysine; 11. Arginine; 12. Acetate; 13. Proline; 14. N-acetyl-Groups; 15. Methionine; 16. Glutamine; 17. Acetone; 18. Glutamate; 19.Pyruvate; 20. Pyroglutamate; 21. Citrate; 22. Dimethylamine; 23. Aspartate; 24. Asparagine; 25. Creatine; 26. Creatine phosphate; 27. Creatinine; 28: Ornithine; 29. Choline; 30. Glucose; 31. Betaine; 32.TMAO; 33. Glycine; 34. Glycerol; 35. Serine; 36. Fructose; 37. Myo-Inositol; 38. Mannose; 39.Tyrosine: 40. Histidine; 41. Phenylalanine; 42. Tryptophan; 43. τ methyl-Histidine; 44. Formate.

PANS *vs* Controls: Gender Analysis

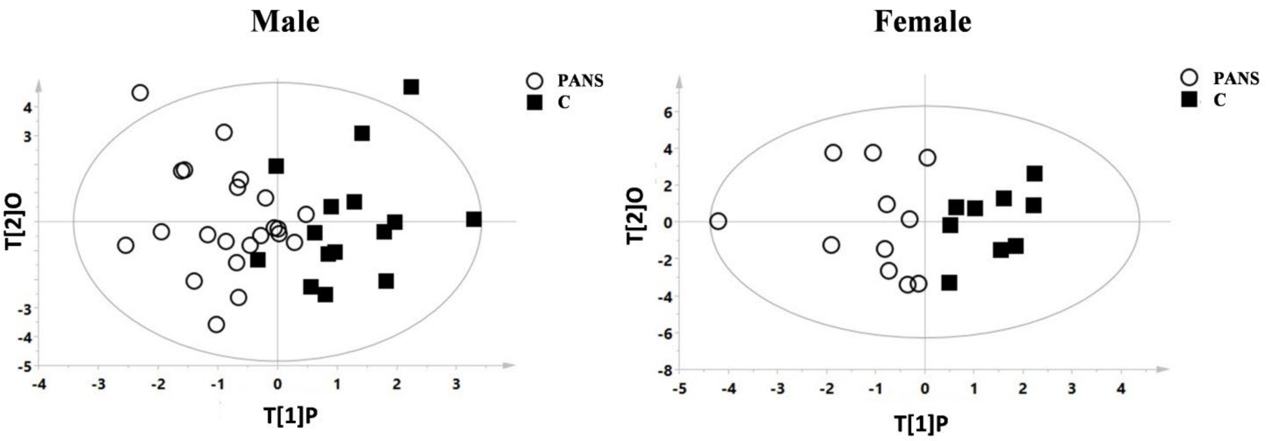

Figure 2S. Multivariate statistical model of the patients affected by PANS and controls subjects based on the gender. Statistical parameters were: male model: $R^2X=0.40$, $R^2Y=0.57$, $Q^2=0.41$, *p*-value = 0.008. Female model: $R^2X=0.6$, $R^2Y=0.6$, $Q^2=0.30$, *p*-value = 0.06.
